# Correlation of C-reactive Protein, Lactate Dehydrogenase, and Serum Calcium With the CT Severity Index in Acute Pancreatitis

**DOI:** 10.7759/cureus.110456

**Published:** 2026-06-08

**Authors:** Kumaresh Pandian, Vineeth Vishwanath, Balaji Singh, Arya Sivakumaran

**Affiliations:** 1 General Surgery, Sri Ramachandra Institute of Higher Education and Research, Chennai, IND

**Keywords:** acute pancreatitis, c-reactive protein, ct severity index, inflammatory biomarkers, lactate dehydrogenase, serum calcium, severity prediction

## Abstract

Background

Acute pancreatitis exhibits a wide clinical spectrum ranging from mild, self-limiting disease to severe forms associated with significant morbidity and mortality. Early identification of disease severity is crucial for guiding management and triaging patients who require close monitoring or intensive care. Biochemical markers that are simple and readily available may aid in early severity prediction.

Methodology

This prospective study included 55 adult patients diagnosed with acute pancreatitis and treated in the Department of General Surgery at Sri Ramachandra Institute of Higher Education and Research between November 2020 and November 2021. Patients with chronic pancreatitis, recurrent pancreatitis, or pancreatic malignancy were excluded. All patients underwent biochemical evaluation including C-reactive protein (CRP), lactate dehydrogenase (LDH), and serum calcium, along with contrast-enhanced CT of the abdomen using a pancreatic protocol. The CT severity index (CTSI) was calculated, and correlations with biochemical parameters were analyzed.

Results

Of the 55 patients, alcohol was the etiology in 40 (72.7%) patients and gallstones in 15 (27.3%) patients. A statistically significant correlation was observed between CRP levels and the CTSI, as well as between LDH levels and the CTSI. No statistically significant correlation was found between serum calcium levels and the CTSI.

Conclusions

CRP and LDH are useful biochemical markers for predicting the severity of acute pancreatitis when correlated with the CTSI. Serum calcium did not demonstrate a significant association with disease severity in this study. These markers may assist in early risk stratification, although larger multicenter studies are required to validate these findings.

## Introduction

Acute pancreatitis is an acute inflammatory condition of the pancreas with a highly variable clinical course, ranging from a mild, self-limiting illness to a severe, life-threatening disease associated with significant morbidity and mortality. The global incidence of acute pancreatitis has been increasing, largely due to rising alcohol consumption and gallstone disease [1,2]. While most patients recover with supportive management, approximately 20-30% develop severe acute pancreatitis, which is associated with systemic inflammatory response syndrome, pancreatic necrosis, multiorgan dysfunction, and increased mortality [3].

Severe acute pancreatitis typically follows a biphasic course. The early phase is characterized by an exaggerated inflammatory response that may result in organ failure within the first week, whereas the late phase is associated with local complications such as pancreatic necrosis, infected collections, and sepsis, contributing to further morbidity and mortality [4]. Early identification of patients at risk for severe disease is therefore essential to guide aggressive fluid resuscitation, intensive monitoring, and appropriate escalation of care during the critical initial period of illness [5].

Several prognostic scoring systems have been developed to predict disease severity in acute pancreatitis, including Ranson’s criteria, Acute Physiology and Chronic Health Evaluation II, Bedside Index for Severity in Acute Pancreatitis, and the CT severity index (CTSI) [6-8]. Although these scoring systems are widely used, many are complex, require multiple clinical and laboratory parameters, or become reliable only after 48 hours, limiting their usefulness in early clinical decision-making. An ideal prognostic tool should be simple, cost-effective, widely available, and capable of predicting severity early in the disease course.

Biochemical markers such as C-reactive protein (CRP), lactate dehydrogenase (LDH), and serum calcium have been investigated as potential predictors of severity in acute pancreatitis. CRP is an acute-phase reactant that reflects the magnitude of systemic inflammation and has been shown to correlate with pancreatic necrosis and adverse outcomes, particularly when measured after 24-48 hours [9]. LDH, a marker of cellular injury and tissue hypoxia, is included in traditional prognostic scoring systems such as Ranson’s criteria and has been associated with disease severity and mortality [10]. Hypocalcemia has also been linked to severe acute pancreatitis due to fat saponification and systemic inflammatory processes, although its predictive value has shown inconsistent results across studies [11].

Contrast-enhanced CT is central to the assessment of disease severity and local complications in acute pancreatitis. The CTSI combines morphologic pancreatic changes and the extent of necrosis and has demonstrated good correlation with morbidity and mortality [12,13]. However, reliance on imaging alone may delay early risk stratification. Correlating readily available biochemical markers with CTSI may therefore provide a practical and accessible approach to early severity prediction, especially in resource-limited settings. This study aims to evaluate the relationship between CRP, LDH, and serum calcium levels and the CTSI in patients with acute pancreatitis and to assess their utility as individual predictors of disease severity.

## Materials and methods

Study design and setting

This was a prospective observational study conducted in the Department of General Surgery at Sri Ramachandra Institute of Higher Education and Research (Deemed to be University), Chennai, India, over a period of one year from November 2020 to November 2021.

Study population

A total of 55 adult patients aged 18 years and above diagnosed with acute pancreatitis were included in the study. Acute pancreatitis was diagnosed based on clinical presentation supported by elevated serum amylase and/or lipase levels and radiological findings. Patients with chronic pancreatitis, recurrent pancreatitis, or pancreatic malignancy were excluded from the study.

Ethical approval and consent

The study was approved by the Institutional Research Ethics Committee of Sri Ramachandra Institute of Higher Education and Research (IEC reference number: CSP-MED/21/MAR/67/57). Written informed consent was obtained from all participants before enrollment, and the study was conducted in accordance with the Declaration of Helsinki and Indian Council of Medical Research guidelines.

Data collection and clinical assessment

Upon admission, demographic details including age and sex were recorded, along with clinical history and etiology of acute pancreatitis. All patients underwent biochemical evaluation including serum C-reactive protein (CRP), lactate dehydrogenase (LDH), and serum calcium levels. Blood samples were collected during the early course of hospitalization as part of routine clinical evaluation.

Radiological assessment

All patients underwent contrast-enhanced CT of the abdomen using a pancreatic protocol. Contrast-enhanced CT scans were performed according to our institutional protocol at approximately 72 hours after symptom onset/admission. The CTSI was calculated based on pancreatic morphology and the extent of pancreatic necrosis. Based on CTSI scores, patients were categorised into mild, moderate, or severe acute pancreatitis.

Correlation of biochemical markers and CT severity index

The biochemical parameters (CRP, LDH, and serum calcium) were correlated with the CTSI to assess their association with disease severity. Subgroup analysis was also performed based on etiology, including alcohol-related and gallstone-related acute pancreatitis.

Statistical analysis

Statistical analysis was performed using SPSS Statistics for Windows, Version 23.0 (IBM Corp., Armonk, NY, USA). Categorical variables were expressed as frequencies and percentages, while continuous variables were expressed as mean and standard deviation. Pearson’s correlation coefficient was used to assess the relationship between biochemical markers and the CTSI. A p-value of less than 0.05 was considered statistically significant.

## Results

A total of 55 patients diagnosed with acute pancreatitis were included in the study. The mean age of the study population was 38.95 years, with the majority of patients (80%) being below 50 years of age. Overall, 19 (34.5%) patients were aged ≤30 years, 14 (25.5%) were between 31 and 40 years, 11 (20.0%) were between 41 and 50 years, five (9.1%) were between 51 and 60 years, and six (10.9%) were older than 60 years (Table 1). Males constituted the majority of the study population, accounting for 46 (83.6%) patients, while nine (16.4%) patients were female (Table 2).

**Table 1:** Age distribution.

Age	Frequency	Percent
Up to 30 years	19	34.5
31–40 years	14	25.5
41–50 years	11	20.0
51–60 years	5	9.1
Above 60 years	6	10.9
Total	55	100.0

**Table 2:** Gender distribution.

Gender	Frequency	Percent
Female	9	16.4
Male	46	83.6
Total	55	100. 0

Alcohol was identified as the most common etiology of acute pancreatitis, observed in 40 (72.7%) patients, followed by gallstone disease in 15 (27.3%) patients (Table 3). Based on the CTSI, four patients were classified as having mild acute pancreatitis (CTSI = 0-2), 29 patients had moderate disease (CTSI = 4-6), and 22 patients had severe acute pancreatitis (CTSI = 8-10). The mean CTSI score for the study population was 6.51 (Table 4).

**Table 3:** Etiology.

Etiology	Frequency	Percent
Alcohol	40	72.7
Gallstones	15	27.3
Total	55	100. 0

**Table 4:** CT severity index.

CT severity index	Number of patients
Mild (0–3)	4
Moderate (4–6)	29
Severe (7–10)	22

**Figure 1:**
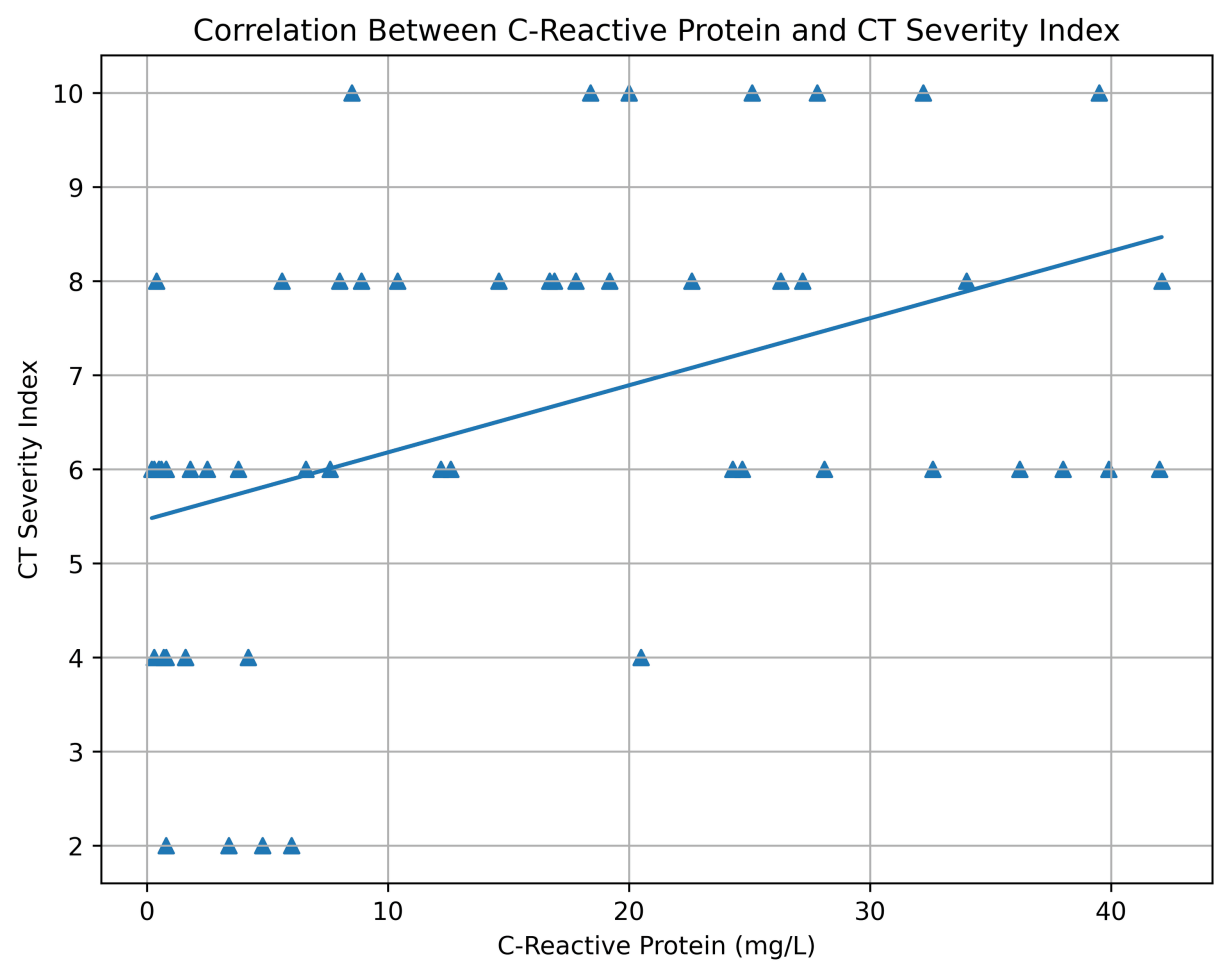
Correlation of C-reactive protein and CT severity index.

**Figure 2:**
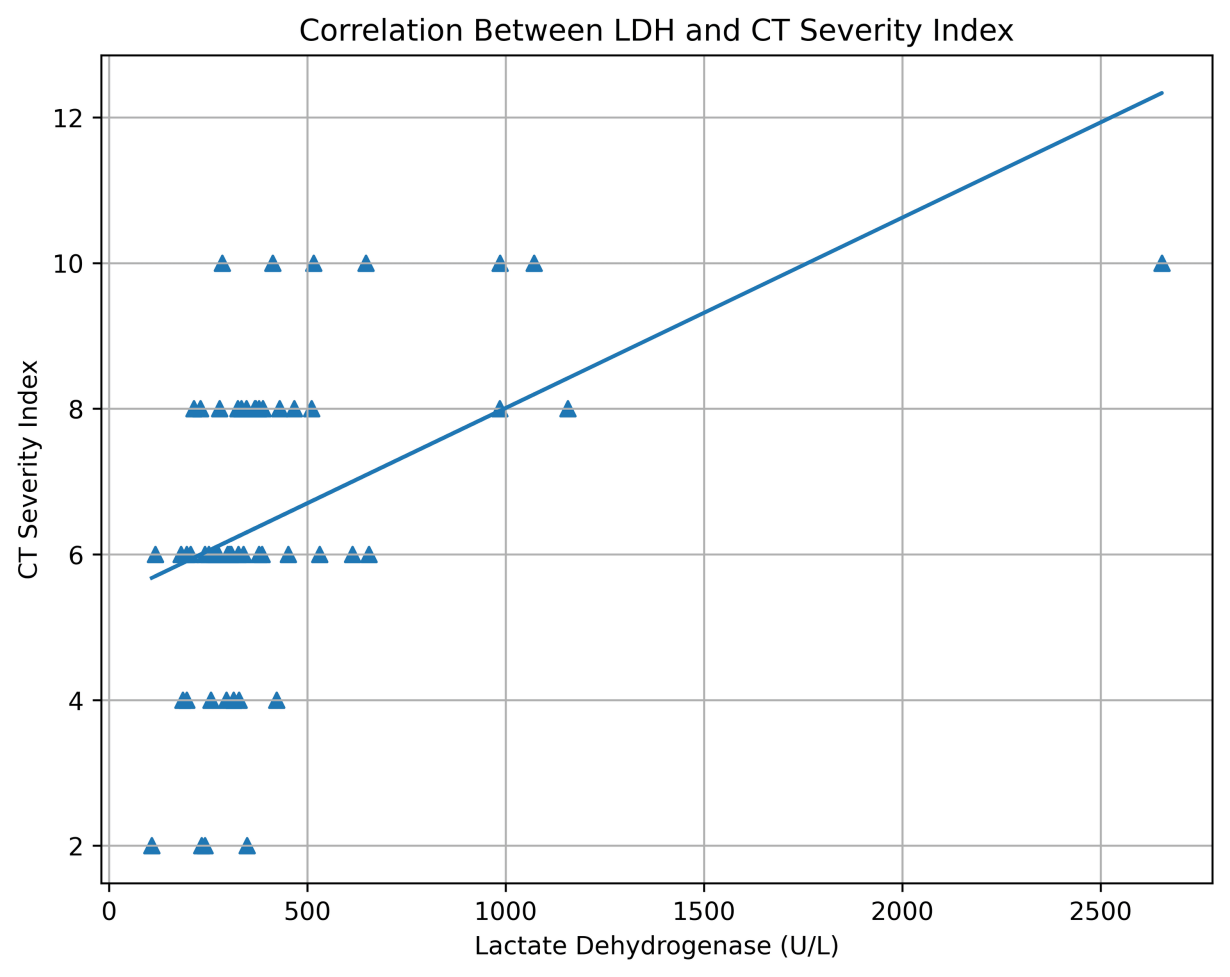
Correlation of lactate dehydrogenase and CT severity index.

**Figure 3:**
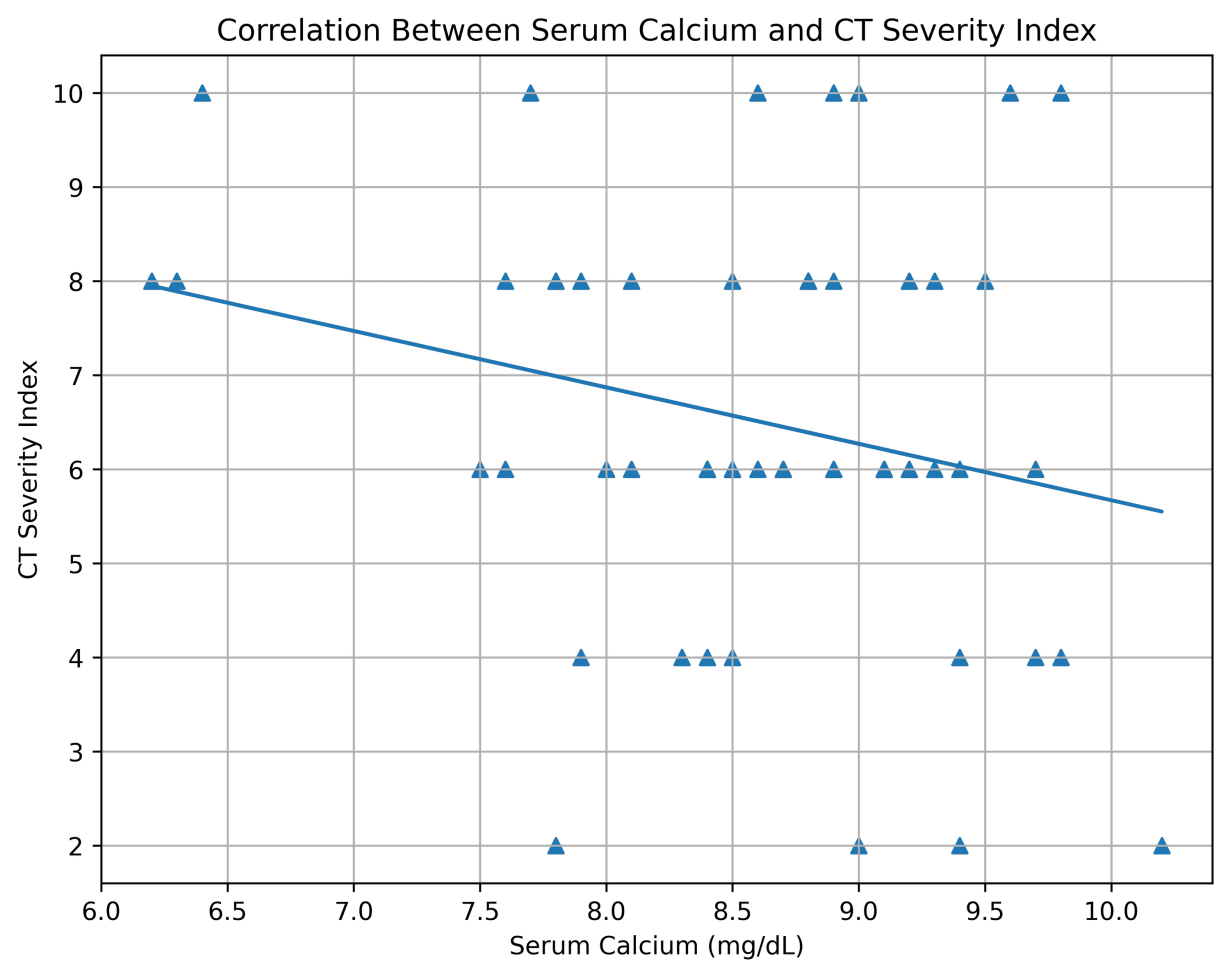
Correlation of serum calcium and CT severity index.

Pearson correlation analysis demonstrated a significant positive correlation between serum CRP levels and CTSI (r = 0.34, p = 0.01), as shown in Figure 1. Similarly, LDH levels also showed a significant positive correlation with CTSI (r = 0.47, p = 0.0005), indicating increasing disease severity, as shown in Figure 2. In contrast, serum calcium levels did not demonstrate a statistically significant correlation with the CTSI (r = -0.03, p = 0.81), as shown in Figure 3.

Subgroup analysis based on etiology revealed that among patients with alcohol-related acute pancreatitis, both CRP and LDH showed statistically significant correlations with the CTSI (p = 0.004 and p = 0.001, respectively). Serum calcium levels in this subgroup did not demonstrate a significant correlation with CTSI (p = 0.074). In patients with gallstone-related acute pancreatitis, none of the biochemical parameters, CRP, LDH, or serum calcium, showed a statistically significant correlation with the CTSI (p = 0.066, p = 0.222, and p = 0.842, respectively).

## Discussion

Principal findings

This prospective observational study evaluated the association between CRP, LDH, and serum calcium with the CTSI in patients with acute pancreatitis. The principal findings demonstrate that CRP and LDH showed statistically significant positive correlations with CTSI, indicating their utility as predictors of radiological severity. In contrast, serum calcium did not show a significant association with disease severity in this cohort.

Interpretation in the context of existing literature

CRP is a widely used inflammatory biomarker and reflects the magnitude of the systemic inflammatory response. In the present study, CRP showed a significant correlation with CTSI (p = 0.01). This finding is consistent with the study by Cho et al., who demonstrated that CRP measured at 24 hours after admission correlated significantly with CT-based severity, although CRP measured at admission did not show a significant association [14]. This highlights the importance of timing in CRP assessment. Conversely, Vengadakrishnan and Koushik did not observe a significant correlation between CRP and CTSI but reported an association with morbidity and mortality, suggesting that CRP may better reflect systemic disease burden rather than radiological severity alone [15].

LDH demonstrated a strong and statistically significant correlation with CTSI in the present study (p = 0.0005). LDH is a marker of cellular injury and hypoxia and is included in established prognostic systems such as Ranson’s criteria. The observed association supports its role as an indicator of severe pancreatic injury. While Vengadakrishnan and Koushik did not find a statistically significant correlation between LDH and CTSI, elevated LDH levels were associated with increased morbidity and mortality, reinforcing its clinical relevance as a severity marker.

Serum calcium did not show a statistically significant correlation with CTSI in this study. Hypocalcemia in acute pancreatitis is believed to result from fat saponification and inflammatory mediator activity; however, its prognostic value remains inconsistent. Thakur et al. demonstrated a significant association between both total and corrected serum calcium levels and CTSI, suggesting calcium as a predictor of severity [11]. The discrepancy with the present findings may be explained by differences in sample size, disease severity distribution, and the lack of corrected calcium measurements in the current study.

Role of CT severity index

The CTSI is an established radiological tool for assessing disease severity and predicting outcomes in acute pancreatitis. Studies by Bollen et al. and Mortele et al. have shown that CTSI and modified CTSI correlate strongly with morbidity and clinical outcomes. These findings support the use of CTSI as a reference standard for evaluating the prognostic value of biochemical markers, as applied in the present study [16,13].

Clinical implications

Early identification of severe acute pancreatitis is essential for guiding management, triage, and intensive monitoring. Although several multifactorial scoring systems are available, their complexity and delayed applicability limit their routine use. Simple, inexpensive, and widely available biomarkers such as CRP and LDH may provide a practical alternative for early risk stratification, particularly in resource-limited settings.

Limitations

This study has certain limitations. First, the relatively small sample size and single-center design may limit the generalizability of the findings. Second, potential confounding factors were not adjusted for, as multivariable analysis was not performed; therefore, the observed correlations between CRP, LDH, and CTSI represent unadjusted associations and may have been influenced by demographic and clinical variables. Third, the interval between symptom onset and biomarker measurement was not consistently documented, which may have affected the levels of time-dependent inflammatory markers such as CRP. In addition, corrected serum calcium levels were not evaluated. Furthermore, independent associations between biomarkers and disease severity could not be assessed due to the absence of multivariable regression analysis. Finally, clinically relevant outcomes such as organ failure, intensive care unit admission, and mortality were not directly analyzed. Therefore, the findings should be considered exploratory and hypothesis-generating, and larger multicenter prospective studies incorporating standardized biomarker assessment, multivariable analyses, and clinical outcome measures are warranted to validate these observations.

Future directions

Larger multicenter studies with standardized timing of biomarker assessment and inclusion of corrected calcium levels are required to validate these findings and further define the role of individual biochemical markers in predicting the severity of acute pancreatitis.

## Conclusions

This study demonstrates that CRP and LDH are simple, readily available, and cost-effective biochemical markers that correlate significantly with radiological severity, as assessed by the CTSI in acute pancreatitis. In contrast, serum calcium did not show a significant association with disease severity in this cohort. CRP and LDH demonstrated significant positive correlations with the CTSI in patients with acute pancreatitis, suggesting an association with radiological disease severity. However, the predictive performance of these biomarkers was not evaluated in the present study. Further studies incorporating diagnostic accuracy analyses and larger patient populations are required to determine their utility as predictive markers of disease severity. The findings support the potential role of CRP and LDH in early risk stratification and identification of patients who may require closer monitoring and aggressive management.
